# Short-Term Effect of Vermicompost Application on Biological Properties of an Alkaline Soil with High Lime Content from Mediterranean Region of Turkey

**DOI:** 10.1155/2014/395282

**Published:** 2014-08-28

**Authors:** Ilker Uz, Ismail Emrah Tavali

**Affiliations:** Department of Soil Science and Plant Nutrition, Faculty of Agriculture, Akdeniz University, 07070 Antalya, Turkey

## Abstract

This study was conducted to investigate direct short-term impact of vermicompost on some soil biological properties by monitoring changes after addition of vermicompost as compared to farmyard manure in an alkaline soil with high lime content from semiarid Mediterranean region of Turkey. For this purpose, mixtures of soil and organic fertilizers in different doses were incubated under greenhouse condition. Soil samples collected in regular intervals were analyzed for biological parameters including dehydrogenase, *β*-glucosidase, urease, alkaline phosphatase activities, and total number of aerobic mesophilic bacteria. Even though soil dehydrogenase activity appeared to be dose-independent based on overall evaluation, organic amendments were found to elevate dehydrogenase activity when sampling periods are evaluated individually. *β*-glucosidase, urease, alkaline phosphatase activity, and aerobic mesophilic bacterial numbers in vermicompost treatments fluctuated but remained significantly above the control. A slight but statistically significant difference was detected between organic amendments in terms of urease activity. Vermicompost appeared to more significantly increase bacterial number in soil. Clearly, vermicompost has a potential to be used as an alternative to farmyard manure to improve and maintain soil biological activity in alkaline calcareous soils from the Mediterranean region of Turkey. Further studies are needed to assess its full potential for these soils.

## 1. Introduction

It is known that microorganisms are the key players in processes such as degradation of organic material, formation of soil organic matter, and nutrient cycles and that these processes are the ones determining soil quality and fertility. Therefore, application of organic fertilizers is a recommended management practice since it stimulates microbial growth and activity leading to chemically and physically more favorable soil environment for plant growth. Microorganisms perform these processes through extracellular enzymes that they secret. Extracellular enzymes could remain active in soil for a long time and they tend to increase with application of organic fertilizers [1]. They contain beneficial microorganisms secreting extracellular enzymes to release nutrients bound to organic compounds. Due to the fact that organic fertilizers include compounds that are substrates for soil enzymes, they also stimulate indigenous microorganisms to perform these processes. Therefore, enzyme activity analyses can be used in order to assess effect of organic amendments on microbial status of a soil. In larger context, soil enzyme activities have been used as indicators of soil quality due to their sensitivity to any changes that may occur in soil [2, 3]. For the last four decades, effect of numerous factors, including organic amendments, on soil enzyme activity has been intensively studied by many scientists [1, 4–11]. It is known that measuring activity of a single soil enzyme is not sufficient since they are generally substrate-specific [12, 13]. For this reason, activities of several enzymes, such as dehydrogenase, *β*-glucosidase, urease, and alkaline phosphatase, are analyzed in such studies [3, 14].

Conventional organic fertilizers, such as compost and farmyard manure, are widely recommended for agricultural production as nutrient source and soil conditioner. In recent years, vermicompost has been considered as an alternative to conventional organic fertilizers. Vermicompost is a product of nonthermophilic biodegradation of organic material by earthworms with the help of microorganisms [15]. Besides being nutrient source and improving soil chemical and physical properties, vermicompost has been reported to contain plant growth promoting compounds (hormones) and to have disease suppression properties, distinguishing it from other conventional organic fertilizers [16]. It has also been suggested that nutrients are released more gradually from vermicompost preventing problems, such as nutrient loss, toxicity, and salinity, which may otherwise be associated with utilization of organic materials under certain conditions [17–19].

Studies conducted on vermicompost have been mainly focused on its effects on plant growth and yield [20–26], its disease suppression properties [27–30], and also changes in microbial activity during the vermicomposting process [15, 31–36]. Several studies investigating relationships between vermicompost and microbial activity in soil under various conditions are also available in scientific literature [37–42]. However, most of these studies were conducted on soils with neutral or acidic pH. Surprisingly, there is limited information on effect of vermicompost on soil biological properties such as soil enzyme activities and relationships with other soil properties in alkaline soils. Moreover, most of the vermicompost-related studies employing measurement of soil microbial activity have been conducted in the presence of plants and with soil samples taken in limited frequency. Even though the ultimate goal is to utilize vermicompost to improve plant growth and yield, one must also know the direct effect of such materials on soil microorganisms without any interference that may come from plants. In addition, such studies must involve more frequent soil sampling in order to monitor changes in microbial activity after addition of vermicompost to soil. Therefore, studies are needed to address these issues. This is especially important for Turkish Mediterranean region because soils of this region are typically in alkaline character and have high lime content and, to our knowledge, no such study involving vermicompost has been conducted in this region. The objective of the research reported here was to investigate direct short-term impact of vermicompost on some biological properties such as bacterial number and soil enzyme activities (dehydrogenase, *β*-glucosidase, urease, and alkaline phosphatase) and some chemical properties by monitoring changes after addition of vermicompost as compared to farmyard manure and to determine relationships existing between measured parameters in an alkaline soils with high lime content from semiarid Mediterranean region of Turkey.

## 2. Materials and Methods

This study was conducted as a pot experiment in which mixtures of soil and organic materials were incubated for sixteen weeks under greenhouse conditions in the Akdeniz University campus. The soil used in the experiment was obtained from a land that was previously used as citrus orchard in Bogacay section of Antalya located in the Mediterranean region of Turkey and taxonomically determined to be fluvent class. The vermicompost was produced mainly from farmyard manure and provided by a local company, and farmyard manure was obtained from the dairy farm belonging to the Faculty of Agriculture at Akdeniz University. Physical and chemical properties of soil, vermicompost, and farmyard manure used in the study are given in Table 1.

The experiment included two organic materials (vermicompost and farmyard manure) applied in five different doses (0, 10, 20, 30, and 40 t ha^−1^ dry weight) and was conducted with randomized factorial block design with four replicates. There were a total of 36 pots. No organic material was added to the control treatments. During the incubation period, soil moisture was kept at 60% of the field capacity water content. Soil samples for each pot were collected in regular intervals (0, 1st, 4th, 7th, 10th, 13th, and 16th week) and analyzed for dehydrogenase, *β*-glucosidase, urease, and alkaline phosphatase activities and also total number of aerobic mesophilic bacteria, pH, and EC. At the end of the experiment, organic matter, total nitrogen, and available phosphorus contents in soil were also determined. Moisture content for each soil sample was determined in order to be included in calculations. Soil enzyme activities were measured as described previously [43]. Total number of aerobic mesophilic bacteria was determined by using the dilution plate count method and expressed as cfu g^−1^ dry weight soil [44]. pH and EC were measured in 1 : 2.5 soil-water mixture [45, 46]. Soil organic matter content was measured by using modified Walkley-Black method [47] and total nitrogen by modified Kjheldahl method [48]. Available phosphorus contents of soil samples were analyzed as described by Olsen and Sommers [49].

Statistical analysis including repeated measures ANOVA, Duncan multiple range test, and Pearson correlation test were conducted using SPSS software version 17.0 [50].

## 3. Results and Discussion

### 3.1. Organic Matter

Organic matter contents of the soil samples at the end of the incubation period are given in Table 2. Initial organic matter content of the soil was 2.1% before the addition of organic materials and this value changed depending on application doses and time. At the end of the experiment the highest organic matter value was obtained with farmyard manure applied in 40 t ha^−1^ dose and the lowest value was with the control treatment. This difference was found to be statistically significant (*P* < 0.001). Also, both organic materials increased the organic matter content depending on their application rate compared to the control. However, no significant difference was observed between organic materials applied in the same doses. According to the correlation analysis, in vermicompost and farmyard manure-amended soils there was a positive relationship between organic matter and urease activity (*P* < 0.01) (*r* = 0.653 and *r* = 0.576, resp.) and a positive relationship between organic matter and total nitrogen content (*P* < 0.01) (*r* = 0.795 and *r* = 0.766, resp.). Also, in vermicompost treatments, there appeared to be a positive relationship between organic matter and dehydrogenase activity (*P* < 0.05, *r* = 0.472) and total number of aerobic mesophilic bacteria (*P* < 0.01, *r* = 0.734).

The data indicate that vermicompost application has significant effect on soil organic matter and this effect is similar to farmyard manure suggesting that vermicompost can be considered as a good alternative to farmyard manure to improve soil organic matter. Several reports support this conclusion and indicate that vermicompost improves soil physical and chemical properties by providing humus to soil [17–19]. According to a previously published report, vermicompost produced from pig manure also increases soil organic matter as well as total nitrogen, phosphorus, potassium, calcium, zinc, and manganese and decreases pH and bulk density [51]. Another study revealed that soil organic matter has significant relations with some of the soil properties such as soil urease and dehydrogenase activities and bacterial numbers [12]. Our data showing positive correlation between soil organic matter and total nitrogen, urease and dehydrogenase activities, and total number of aerobic mesophilic bacteria are in agreement with this report.

### 3.2. Total Nitrogen

Total nitrogen content of the soil before the experiment was measured to be 0.09%. At the end of the incubation period, total nitrogen values ranged from 0.1% (the control) to 0.29% (30 and 40 t ha^−1^ vermicompost) and this change was found to be significant (*P* < 0.001) (Table 2). Organic material applications significantly increased the total nitrogen content in soil compared to the control. However, no significant difference was found between organic materials applied in the same doses. According to the correlation analysis, there were positive relationships between total nitrogen content and urease activity (*P* < 0.01, *r* = 0.904, and *r* = 0.822) and total number of aerobic mesophilic bacteria (*P* < 0.01, *r* = 0.876, and *r* = 0.607) in soils treated with vermicompost and farmyard manure, respectively.

Organic matter is the major source of nitrogen in soil. When organic fertilizers are added to soil in order to fill organic matter storage, nitrogen is one of the major nutrients supplied to soil. Therefore, in our study, it was not surprising to observe that vermicompost and farmyard manure significantly elevated the nitrogen content of soil. Even though total nitrogen content of vermicompost that we used was higher than that of farmyard manure (Table 1), total nitrogen content at the end of the incubation period was similar in soils treated with organic materials in the same doses. It was found that some of organic nitrogenous compounds in organic materials are converted to nitrogen and that this nitrogen is generally released slowly [52]. This may explain the similarity in soil nitrogen contents in vermicompost and farmyard manure treatments. It was reported that total nitrogen content of vermicompost is in relation with microbial number and urease activity in vermicompost and nitrogen serves as substrate for urease [53]. This relationship may also be seen in soils after vermicompost application and explain positive correlations that we observed between total nitrogen and urease activity and total number of aerobic mesophilic bacteria in our study.

### 3.3. Available Phosphorus

Initial available phosphorus content of the soil was 30 ppm. At the end of the experiment, depending on time and application doses, the values varied between 38 ppm and 109.25 ppm (Table 2). The highest available phosphorus value was obtained with 40 t ha^−1^ vermicompost application and the lowest with the control. The difference among treatments was found to be significant (*P* < 0.001). Available phosphorus appeared to be elevated in all treatments, including the control. However, no significant difference was observed between vermicompost and farmyard manure applied in the same doses (except 20 t ha^−1^ treatment). Correlation analysis based on values at the 16th week indicated that in soils treated with vermicompost, there was a positive relationship between available phosphorus and alkaline phosphatase activity (*P* < 0.05, *r* = 0.536). In farmyard manure treatments, a positive relationship was observed between available phosphorus and pH (*P* < 0.05, *r* = 0.479).

Vermicompost and farmyard manure applied to soil significantly increased available phosphorus content of soil compared to the control treatment. However, no significant difference was observed between these two organic materials applied in the same doses in terms of available phosphorus even though phosphorus content of vermicompost used in our study is two times higher than that of farmyard manure. This situation may be attributed to the fact that phosphorus in vermicompost is released more gradually [17–19]. On the other hand, a significant positive relationship was observed between available phosphorus and alkaline phosphatase activity only in soils treated with vermicompost in our study. This result is in agreement with the observation previously reported [54].

### 3.4. pH and EC (Electrical Conductivity)

**Figure 1:**
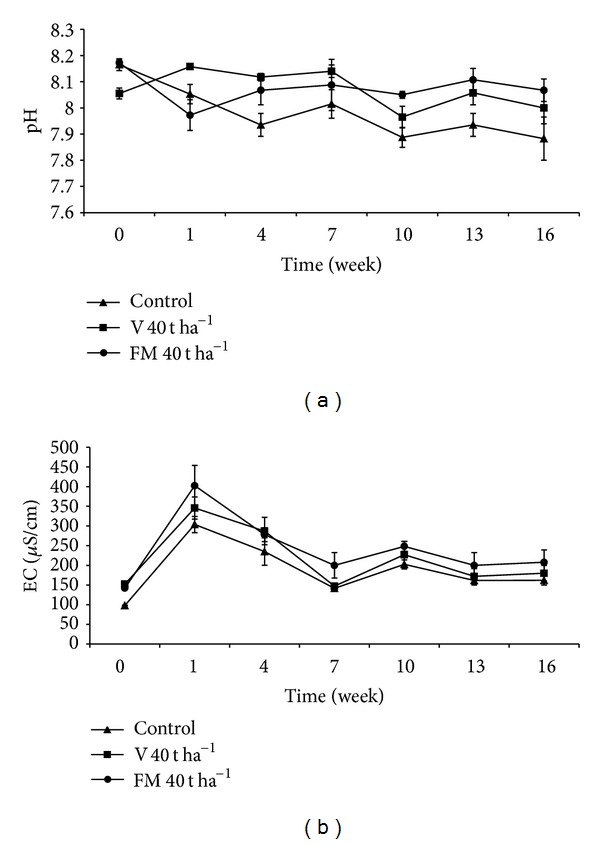
Changes in pH (a) and EC (b) in soils treated with vermicompost (V) and farmyard manure (FM) at the rate of 40 t ha^−1^. Error bars represent standard errors based on four replicates.

Changes that occurred in pH in 40 t ha^−1^ treatment during the incubation period are given in Figure 1(a). The pH values in organic material-amended soils showed similar trend during the experiment. In general, soils with organic materials showed higher pH values compared to the control treatment and based on calculated mean values, this difference was found to be statistically significant (*P* < 0.001) (Table 3). Vermicompost applied in 30 t ha^−1^ dose resulted in higher pH values in fourth, seventh, thirteenth, and sixteenth weeks compared to farmyard manure applied in the same dose (data not shown). The lowest soil pH was recorded with the control treatment in the sixteenth week (*P* < 0.001). Also, there appeared to be a significant effect of treatment *X* time interaction on soil pH (*P* < 0.05).

Even though EC values of soils in all treatments fluctuated during the experiment there was an increasing trend in general (Figure 1(b)). Except for the 10 t ha^−1^ treatment, all other treatments resulted in increased EC values compared to the control treatment. The highest EC value was observed in the first week with farmyard manure applied in 40 t ha^−1^ dose and significantly differed from the control soil (*P* < 0.001). In general, farmyard manure treatments show higher EC values than vermicompost treatments during the incubation. However, overall difference among treatments was not significant based on calculated mean values (Table 3). Effect of treatment *X* time interaction on soil EC was also statistically insignificant.

Soil pH is an important soil property that has direct impact on plant growth, availability of nutrients, and microbial activity. It is generally thought that applications of organic materials reduce soil pH. However, contrary to the general belief, we observed that soil pH in treatments with organic materials generally remained above the control levels during the incubation period. Similar results were also reported by previous studies [38, 55]. This may be due to the fact that organic materials used in our study have alkaline reaction (Table 1). Indeed, some researchers determined that organic fertilizers with high pH may not lower soil pH [56–58]. Applications of organic fertilizers are known to increase soil EC and our study showed an increase in EC by vermicompost and farmyard manure. However, only the farmyard manure, but not the vermicompost, significantly increased the EC compared to the control in the first week. This difference between these organic materials may be due to the gradual release of nutrients from vermicompost [17–19]. The increase in EC values did not occur in a level to cause any salinity problem. Several researchers obtained similar results and concluded that, in general, organic fertilizers do not cause salinity problem when applied in moderate levels [59–61].

### 3.5. Dehydrogenase Activity

**Figure 2:**
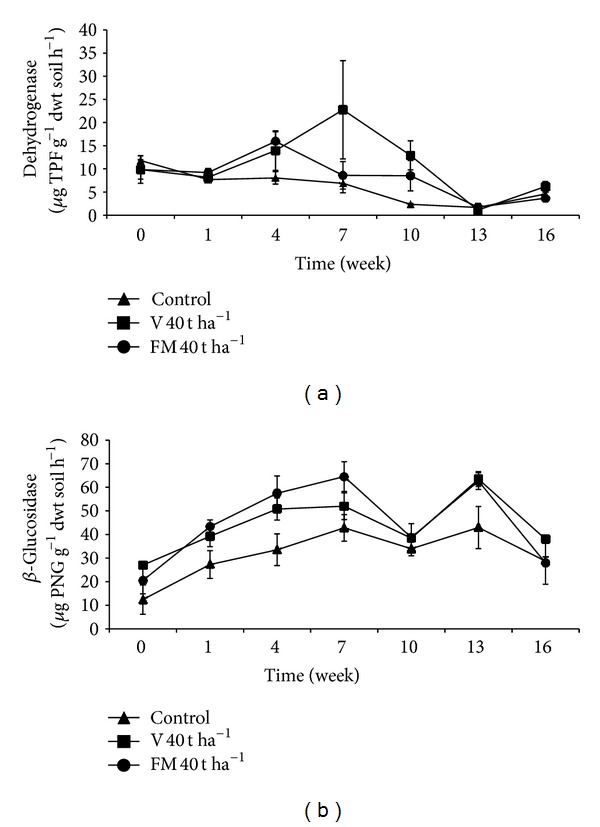
Changes in dehydrogenase (a) and *β*-glucosidase (b) activity in soils treated with vermicompost (V) and farmyard manure (FM) at the rate of 40 t ha^−1^. Error bars represent standard errors based on four replicates.

Changes that occurred in soils dehydrogenase activity in 40 t ha^−1^ treatments during the incubation period are given in Figure 2(a). In soil receiving organic materials, dehydrogenase activity started to increase from the first week to the 4th–7th weeks of the experiment and then decreased to control levels at around 10th–13th weeks. The control treatment, however, showed gradual decrease from the beginning of the experiment. The highest dehydrogenase activity was observed in the 7th week in soils receiving organic materials and significantly differed from control treatments (*P* < 0.001). However, repeated measure ANOVA and subsequent statistical analysis revealed that overall difference among treatments was not significant based on calculated mean values (Table 3). Effect of treatment *X* time interaction on soil dehydrogenase activity was also statistically insignificant.

It is possible to assess overall soil microbial activity by measuring activity of dehydrogenase which is an intracellular enzyme that reflects oxidative activity of microflora [62, 63]. In the present study, vermicompost and farmyard manure applications resulted in elevated dehydrogenase activity compared to the control treatment when results from each sampling period are evaluated individually even though overall no significant difference was detected among treatments. Elevated dehydrogenase activity is possibly due to utilization of nutrients provided by the organic materials by microorganisms resulting in an increase in microbial activity. Similarly, several researchers reported that organic fertilizers increase soil dehydrogenase activity [64–66]. In addition, vermicomposts produced from various organic materials are known to have high dehydrogenase activity and to increase soil dehydrogenase activity when added to soil [67–71]. Our data showed that, after the 4th–7th weeks, dehydrogenase activity begins to decrease toward control level. This trend was also observed in a previous study [72]. This may be due to accumulation of nitrification products (NO_3_^−^ and NO_2_^−^) or some other compounds that have inhibitory effect on dehydrogenase [73, 74]. On the other hand, an alternative and perhaps the most likely reason is the depletion of easily degradable compounds supplied by vermicompost and farmyard manure after the 4th–7th weeks causing microorganism to lower their activities.

### 3.6. *β*-Glucosidase Activity

From the beginning of the incubation period, soils with organic materials showed higher *β*-glucosidase activity compared to the control (Figure 2(b)). In particular in soils treated with organic materials in highest doses (30 and 40 t ha^−1^) the difference was more prominent in the period of 0–10th weeks. Overall difference between organic material treatments and the control was found to be statistically significant (*P* < 0.01) (Table 3). Farmyard manure appeared to promote *β*-glucosidase activity greater than vermicompost during this period even though the difference between organic materials was not statistically significant based on calculated mean values (Table 3). The highest *β*-glucosidase activity was observed in soils receiving 30 t ha^−1^ vermicompost in 13th week (*P* < 0.001). No significant effect of treatment* X* time interaction on *β*-glucosidase activity was detected. Correlation analysis indicated that there were positive relationships between soil *β*-glucosidase activity and urease activity in vermicompost and farmyard manure treatments (*P* < 0.01, *r* = 0.347, and *r* = 0.231, resp.) and positive relations between *β*-glucosidase and total number of aerobic mesophilic bacteria (*P* < 0.01, *r* = 0.260, and *P* < 0.05, *r* = 0.195, resp.).

*β*-Glucosidase is one of the enzymes that are involved in degradation of cellulose, one of the most abundant carbohydrate (a polysaccharide) in nature, producing glucose which is an important energy source for microorganisms in soil. *β*-Glucosidase, like other extracellular enzymes, is originated from microorganisms [2]. Therefore, activity of this enzyme can be used to assess carbon turnover that has an impact on soil fertility [43]. In our study, both vermicompost and farmyard manure applications resulted in elevated *β*-glucosidase activity compared to the control. This is possibly due to presence of substrates for *β*-glucosidase in organic fertilizers leading to high level of this enzyme and high number of microorganisms capable of secretion of the enzyme. When these organic materials are added to soil, *β*-glucosidase already present in the material remains active in soil and microorganisms added to soil may continue enzyme secretion resulting in elevated *β*-glucosidase activity. Moreover, organic compounds such as cellulose in organic materials could also stimulate indigenous soil microorganisms to produce *β*-glucosidase. Several reports indicated that organic fertilizers increase soil *β*-glucosidase activity depending on carbon composition of materials used in the fertilizer production process [64, 75, 76]. Also, vermicomposts produced from various organic materials are known to have high *β*-glucosidase activity and their applications to soil increase this enzyme's activity in soil [31, 32, 41, 67, 77]. It was reported that, during the vermicomposting process, significant correlations between *β*-glucosidase, urease, and general microbial activity exist [34]. In our study, we observed similar correlations in soil.

### 3.7. Urease Activity

**Figure 3:**
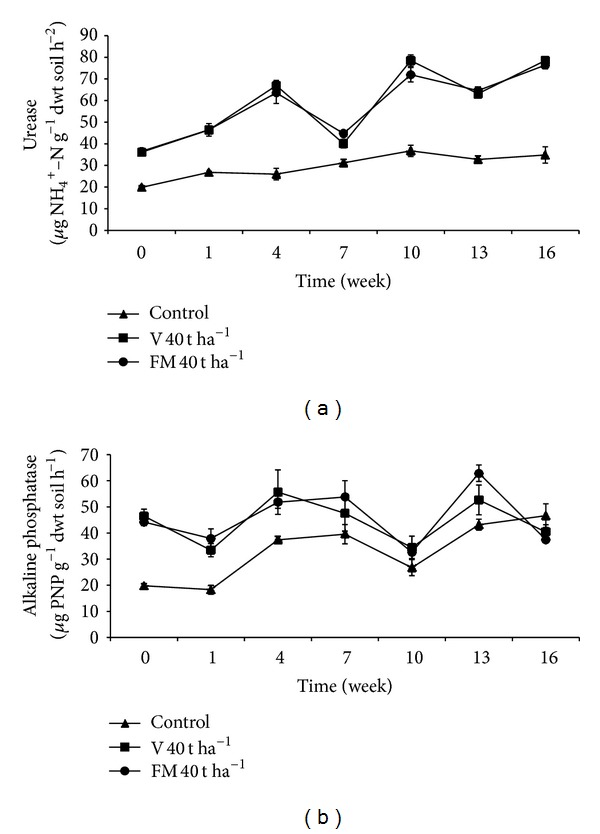
Changes in urease (a) and alkaline phosphatase (b) activity in soils treated with vermicompost (V) and farmyard manure (FM) at the rate of 40 t ha^−1^. Error bars represent standard errors based on four replicates.

Urease activity in soils showed an increasing trend during the incubation period (Figure 3(a)). In general, based on calculated mean values, statistically significant differences exist among treatments (*P* < 0.001) (Table 3). In particular, application of organic materials resulted in significantly higher urease activity compared to the control. The highest urease activity value was observed in soils treated with 10 t ha^−1^ vermicompost in the 10th week (data not shown). Also, statistical analysis revealed that urease activity is significantly affected by the treatment *X* time interaction (*P* < 0.001). According to correlation analysis, there was a positive relationship between urease activity and total number of aerobic mesophilic bacteria in soils treated with vermicompost and farmyard manure (*P* < 0.01, *r* = 0.818, and *r* = 0.709, resp.).

Urease is an extracellular enzyme that is involved in nitrogen cycle and it catalyzes hydrolysis of urea to ammonia [48]. Therefore, it is considered to be an important enzyme since it has a direct effect on soil fertility [2]. In general, urease activity increases with increasing microbial activity in soil [48, 78]. In our study, organic materials added to soil increased soil urease activity compared to the control. This result can be attributed to nitrogenous compounds supplied to soil by organic materials leading to elevated microbial number and activity. In addition, microorganisms and urease already present in the organic fertilizers might also contribute to high soil urease activity. Indeed, urease activity in the treated soils at the beginning of the incubation period, immediately after the addition of the fertilizers, was significantly higher than that of the control. Since the control did not receive any nitrogenous compounds and urease, urease activity remained low and did not fluctuate during the incubation period. It is known that vermicompost increases urease activity in soil [12, 41, 53]. In our study, urease activity in soils treated with vermicompost and farmyard manure showed similar trend during the incubation period even though slight but a statistically significant difference was present. Several studies revealed that soil urease activity is related to urea and urea-like substrates contained in organic materials [64–66, 68, 79]. Therefore, our data may indirectly suggest that vermicompost and farmyard manure used in our study contain similar amount of urea and urea-like substrate. A previously published study showed that factors such as urease and microbial populations that affect soil productivity are directly related to management practices and that these factors correlate with each other when organic fertilizers are applied to soil [71]. This report supports our data showing positive correlation between urease activity and number of aerobic mesophilic bacteria in soils treated with vermicompost.

### 3.8. Alkaline Phosphatase Activity

From the beginning of the experiment, organic material-amended soils had higher alkaline phosphatase activity compared to the control (Figure 3(b)). Even though all treatments, including the control, showed a similar trend during the experiment, organic materials' effect on alkaline phosphatase activity was found to be significant. Based on calculated mean values, organic materials resulted in significantly higher alkaline phosphatase activity compared to the control (*P* < 0.01) (Table 3). On the other hand, no significant difference was observed between organic materials in terms of the enzyme activity. Effect of treatment *X* time interaction on alkaline phosphatase activity was found to be statistically significant (*P* < 0.01). There appeared to be a negative relationship between alkaline phosphatase activity and EC (*P* < 0.01, *r* = −0.251) in vermicompost treatments and a positive relationship between the enzyme activity and pH (*P* < 0.05, *r* = 0.196) in farmyard manure treatments.

Higher initial alkaline phosphatase activity in treated soils immediately after the addition of organic fertilizers indicates high enzyme activity already present in these fertilizers. The observation that no significant difference, in general, exists between alkaline phosphatase activity values in vermicompost and farmyard manure treatments during the incubation period may indicate that alkaline phosphatase activity and potential for stimulation of indigenous soil organisms to produce this enzyme are similar. This result is in agreement with previous studies reporting that vermicompost has high alkaline phosphatase activity and its application elevates this enzyme's activity and available phosphorus content in soils [34, 41, 53, 54, 72, 80–82]. Also, our data showing significant negative relationship between EC and alkaline phosphatase activity in soils with vermicompost is supported by a previous report [75].

### 3.9. Number of Total Aerobic Mesophilic Bacteria

**Figure 4:**
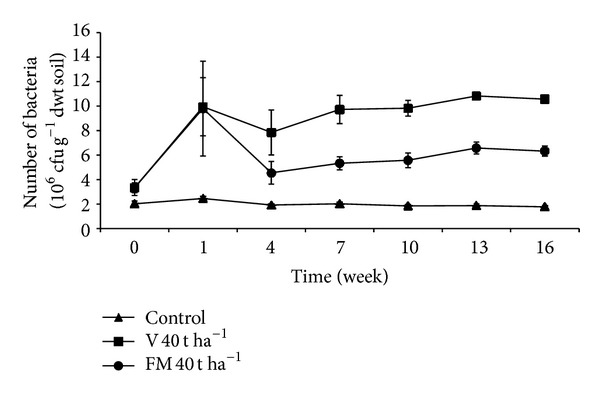
Changes in number of total aerobic mesophilic bacteria in soils treated with vermicompost (V) and farmyard manure (FM) at the rate of 40 t ha^−1^. Error bars represent standard errors based on four replicates.

Aerobic mesophilic bacterial numbers in soils treated with organic materials in 40 t ha^−1^ doses are given in Figure 4. Bacterial numbers immediately increased at the beginning of the experiment in soils treated with organic materials and followed a steady trend after the 4th week. No significant increase was recorded in the control treatment. The difference between organic treatments and the control was found to be statistically significant (*P* < 0.001) (Table 3). Also, in all treatments (except 10 t ha^−1^ dose), vermicompost yielded significantly higher bacterial number in soil compared to farmyard manure (*P* < 0.001). After the 4th week, bacterial numbers in organic treatments remained in slightly increasing trend. Effect of treatment *X* time interaction on microbial number was found to be statistically insignificant.

Soil organisms are the main players in transformation of chemicals in soil. Since they are involved, especially, in transformation of plant nutrients soil microorganisms are the major factor determining soil fertility. In the present study, vermicompost applications resulted in higher number of aerobic mesophilic bacteria in soil compared to farmyard manure. After the application of organic materials to soil, in all doses, slightly higher microbial count in treated soils can be attributed to microorganisms added with organic materials. In the first week, however, microbial number significantly increased in all vermicompost-treated soils. In farmyard manure treated soil, except for 40 t ha^−1^ treatment, there was no sudden increase, but rather smooth increase during the incubation period. In general, soils with vermicompost (except 10 t ha^−1^ application) showed significantly higher bacterial number than soils with farmyard manure and the control. This result is consistent with earlier reports [39]. Even though the difference between microbial numbers in vermicompost and farmyard manure treatments is less than 10-fold, it was statistically significant and constant during the incubation period. This data may imply that vermicompost supports higher microbial population size and, perhaps, greater diversity. Several researchers pointed out high microbial population and diversity in soils resulted from vermicompost applications [33, 34, 41, 83–85].

## 4. Conclusions

Results of this study showed that vermicompost has significant impact on some of soil biological and chemical properties and this effect was generally similar to farmyard manure in the absence of plants in the test soil. However, vermicompost application was found to support higher bacterial number in this soil. Even though soil dehydrogenase activity appeared not to be dose-dependent based on overall evaluation, organic amendments were found to elevate dehydrogenase activity when results from each sampling period are evaluated individually. In terms of parameters investigated in this study, vermicompost has a potential to be used as an alternative to farmyard manure in alkaline soils in Mediterranean region of Turkey. Considering the fact that it also has plant growth promoting compounds and pathogen suppressing properties, vermicompost may provide additional benefits to farmers compared to other conventional organic fertilizers such as farmyard manure. In order to assess its full potential for agricultural sector and to promote its production and utilization in the region, it is necessary to evaluate its long term effects on alkaline soils under regional conditions. These efforts should include more detailed microbial studies, such as microbial community analysis, as warranted by the present study. Also, further studies in the field with various agricultural plants should be conducted.

## Figures and Tables

**Table 1:** Physical and chemical properties of soil and organic materials used in the study.

	Soil	Vermicompost	Farmyard manure
Texture	Clay loam	—	—
pH (1 : 2.5)	7.62	7.80	8.19
EC (1 : 2.5) *µ*S/cm	110	1450	4500
Lime (%)	17.7	—	—
Organic matter (%)	2.1	48.95	67.87
Total N (%)	0.09	1.90	1.49
C/N	13.53	14.94	26.41
P (%)	0.0013	2.05	0.78
K (%)	0.19	0.8	2.56
Ca (%)	0.40	1.89	3.03
Mg (%)	0.09	0.92	0.68
Mn (ppm)	2.67	500	741
Zn (ppm)	0.47	100	52.62
Cu (ppm)	0.25	44	72
Fe (ppm)	1.20	1575	565

**Table 2:** Organic matter, total nitrogen, and available phosphorus contents of soils treated with vermicompost (V) and farmyard manure (FM).

Treatment	Organic matter (%)	Total N (%)	Available P (ppm)
Control	1.92^c^ ^1^	0.10^c^	38.00^d^
V 10 t ha^−1^	2.30^b^	0.25^b^	81.00^c^
V 20 t ha^−1^	2.52^b^	0.28^a^	88.00^b^
V 30 t ha^−1^	2.90^a^	0.29^a^	90.00^b^
V 40 t ha^−1^	3.00^a^	0.29^a^	109.25^a^
FM 10 t ha^−1^	2.32^b^	0.24^b^	79.25^c^
FM 20 t ha^−1^	2.50^b^	0.28^a^	85.00^bc^
FM 30 t ha^−1^	3.05^a^	0.28^a^	88.00^b^
FM 40 t ha^−1^	3.10^a^	0.28^a^	108.25^a^
LSD (5%)	22.16^∗∗∗2^	67.30∗∗∗	107.1∗∗∗

^1^Means in the same column followed by the same letter are not significantly different.

^2∗∗∗^ *P* < 0.001.

**Table 3:** Mean values of parameters measured in regular intervals during the study.

Treatment	pH	EC (*µ*S/cm)	Dehydrogenase (*µ*g TPF g^−1^ dwt h^−1^)	*β*-Glucosidase (*µ*g PNG g^−1^ dwt h^−1^)	Urease (*µ*g NH_4_^+^-N g^−1^ dwt h^−2^)	Alkaline phosphatase (*µ*g PNG g^−1^ dwt h^−1^)	Number of bacteria (10^6^ cfu g^−1^ dwt soil)
Control	7.96^d^ ^1^	186.21	6.14	31.66^b^	29.73^d^	33.08^b^	1.98^d^
V 10 t ha^−1^	7.99^cd^	180.29	9.68	41.13^a^	54.54^c^	44.17^a^	6.16^b^
V 20 t ha^−1^	8.17^a^	195.70	9.30	40.66^a^	54.67^bc^	42.36^a^	9.89^a^
V 30 t ha^−1^	8.14^ab^	214.83	8.73	42.96^a^	56.69^abc^	45.08^a^	6.61^b^
V 40 t ha^−1^	8.07^bc^	215.81	10.70	44.14^a^	58.53^a^	44.37^a^	8.86^a^
FM 10 t ha^−1^	7.99^cd^	219.47	10.23	39.69^a^	57.67^abc^	40.52^a^	5.47^b^
FM 20 t ha^−1^	8.14^ab^	197.56	9.16	41.52^a^	58.37^ab^	46.96^a^	4.88^bc^
FM 30 t ha^−1^	8.09^ab^	200.55	9.84	45.97^a^	56.55^abc^	44.76^a^	3.44^cd^
FM 40 t ha^−1^	8.07^bc^	239.66	8.22	47.65^a^	57.77^abc^	45.80^a^	5.92^b^
LSD (5%)	7.33^∗∗∗2^	NS^3^	NS	3.322∗∗	63.198∗∗∗	4.104∗∗	17.775∗∗∗

^1^Means in the same column followed by the same letter are not significantly different. (LSD; *P* < 0.05).

^2∗∗^ *P* < 0.01.

***P < 0.001.

^3^NS: not significant.
